# Supplementary material for: CURTAINs for your sliding window: Constructing unobserved regions by transforming adjacent intervals
Source: Front Big Data. 2023 Mar 21;6:899345. doi: 10.3389/fdata.2023.899345 (PMC10072325; doi:10.3389/fdata.2023.899345)
Supplement: Supplementary file 1 [file Data_Sheet_1.PDF]

Supplementary Material

1 SUPPLEMENTARY TABLES AND FIGURES

1.1 Tables

Table S1. The performance of the CURTAINs transformer when trained on different amounts of training data. For all models the sidebands, signal region and outer-bands are 200 GeV wide, with the model trained on SB1 ($3200 \leq m_{JJ} < 3400$ GeV) and SB2 ($3600 \leq m_{JJ} < 3800$ GeV). Validation of performance is measured using a classifier trained on the transformed data against the real data in the target region and quantified by the area under the ROC curve. For 100% of the available statistics, there are around 80,000 training examples in SB1 and 45,000 training examples in SB2. In all cases there are no signal events injected into the training sample. The model architecture optimised for the full available training dataset has been used without any optimisation for all levels. The training time scales linearly with the size of the training dataset, with the full available statistics requiring 6 hours to train for 1000 epochs on an Nvidia RTX 3080 GPU. The performance decreases with the available training data, with the outer-bands most impacted. The performance stays around the level of 0.50–0.52 for all transformations down to 25% of the available training statistics, though a noticeable drop in performance is observed between the two side-bands at 50%.

Training statistics	SB1→SB2	SB2→SB1	SB1→OB1	SB2→OB2	SB1→SR ∪ SB2→ SR
100%	0.504	0.504	0.519	0.512	0.509
75%	0.508	0.509	0.518	0.509	0.505
50%	0.518	0.519	0.518	0.521	0.506
25%	0.518	0.520	0.512	0.516	0.505
10%	0.533	0.534	0.527	0.523	0.508
5%	0.530	0.532	0.534	0.527	0.511

1.2 Figures

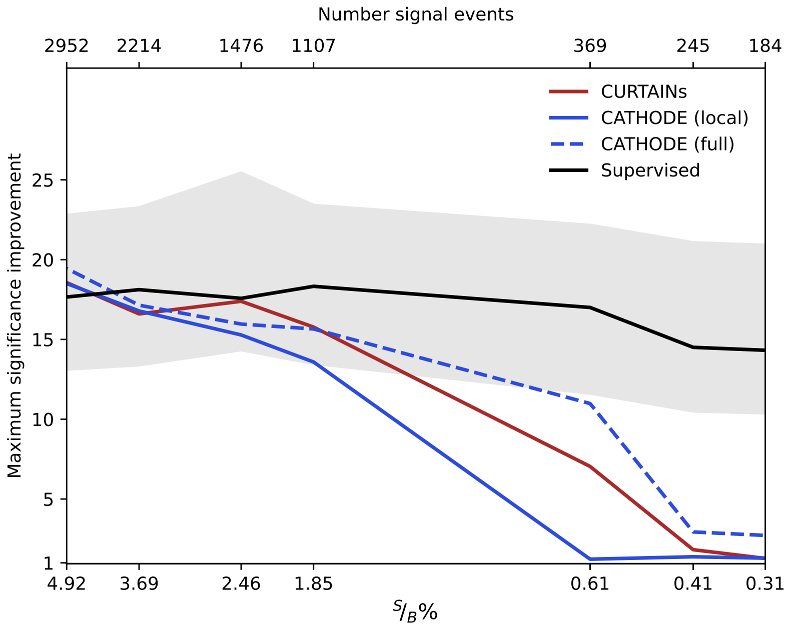

Figure S1. The significance improvement as a function of decreasing signal purity (raw signal events) for the different background template models (CURTAINs - red, CATHODE (local) - solid blue, CATHODE (full) - dashed blue) and a fully supervised classifier (black). All classifiers trained in the signal region $3300 \leq m_{JJ} < 3700$ GeV for varying levels of signal doping. The solid lines show the mean value of fifty classifier trainings with different random seeds. The uncertainty encompasses 68% of the runs either side of the mean.

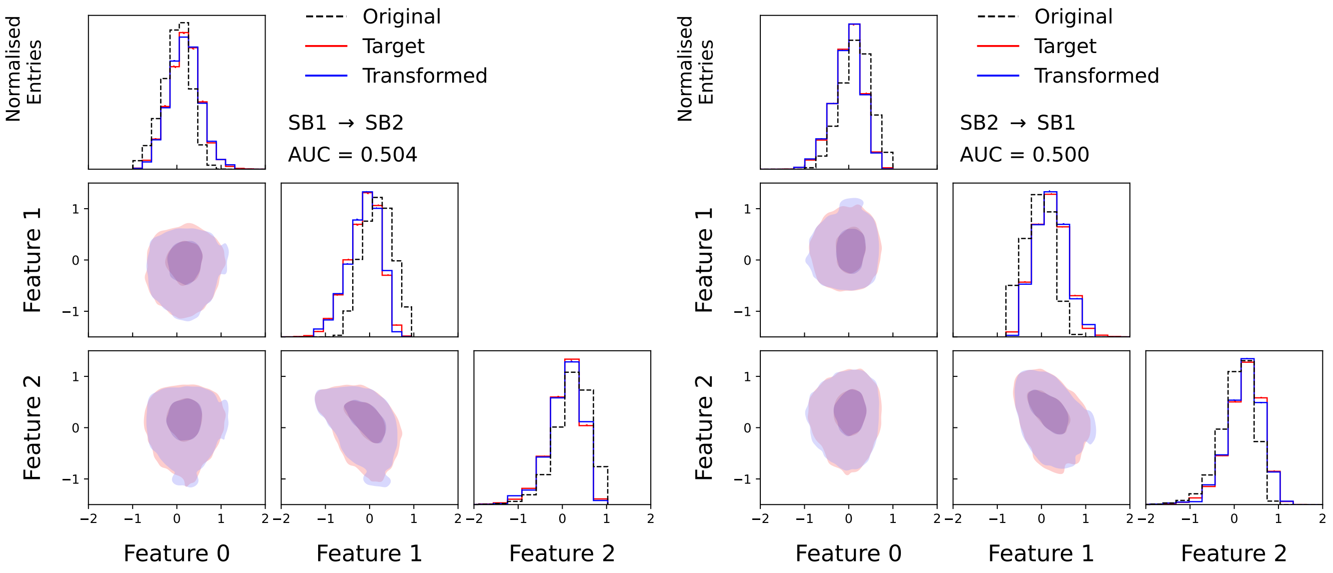

Figure S2. Input, target and transformed data distributions for a toy example with enhanced correlation between the input variables and the invariant mass. Features 0 and 1 are linearly correlated to m_{JJ} whilst and Feature 3 is proportional to the inverse cube of m_{JJ}. In comparison to the architecture optimised for the nominal features, the size of each coupling layer has been increased to four residual blocks, each with two hidden layers of 64 nodes. Distributions are shown for transforming data from SB1 to SB2 (left) and SB2 to SB1 (right), with the model trained on SB1 ($3200 \leq m_{JJ} < 3400$ GeV) and SB2 ($3600 \leq m_{JJ} < 3800$ GeV). The data from SB1 (SB2) is transformed with a forward (inverse) pass of the CURTAINs model into the target region. The diagonal elements show the individual features with the off diagonal elements showing a contour plot between the two observables for the transformed and trained data.

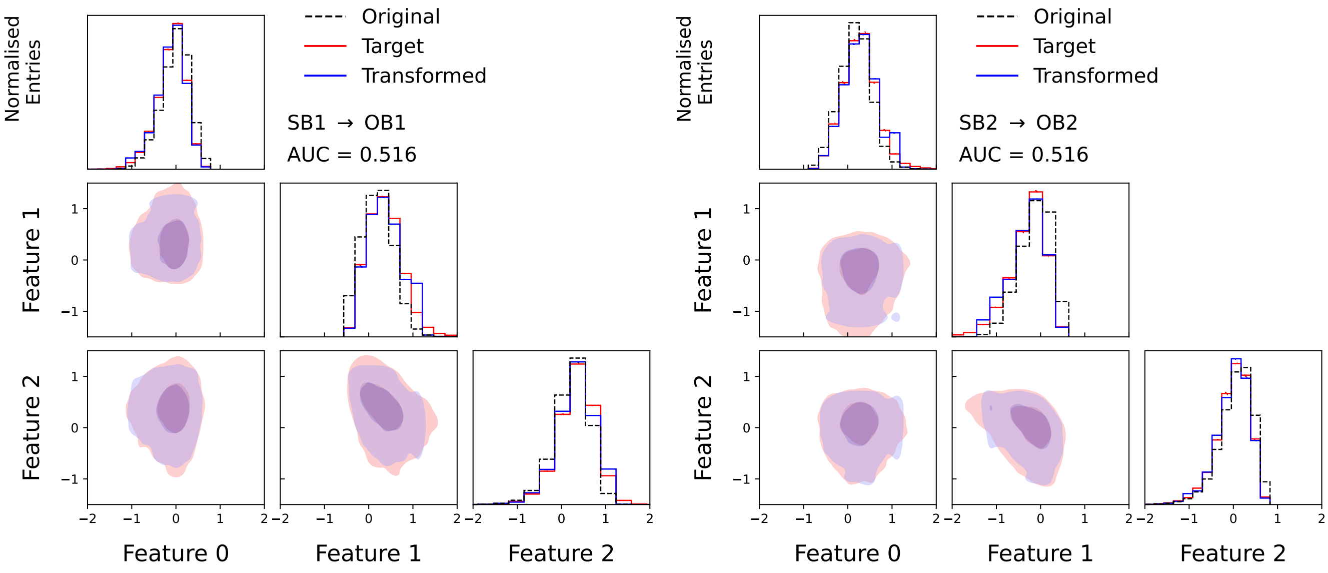

Figure S3. Input, target and transformed data distributions for a toy example with enhanced correlation between the input variables and the invariant mass. Features 0 and 1 are linearly correlated to m_{JJ} whilst and Feature 3 is proportional to the inverse cube of m_{JJ}. In comparison to the architecture optimised for the nominal features, the size of each coupling layer has been increased to four residual blocks, each with two hidden layers of 64 nodes. Distributions are shown for transforming data from SB1 to OB1 (left) and SB2 to OB2 (right), with the model trained on SB1 ($3200 \leq m_{JJ} < 3400$ GeV) and SB2 ($3600 \leq m_{JJ} < 3800$ GeV), with OB1 and OB2 defined as 200 GeV wide windows directly next to SB1 and SB2 away from the signal region. The data from SB1 (SB2) is transformed with an inverse (forward) pass of the CURTAINs model into the target region. The diagonal elements show the individual features with the off diagonal elements showing a contour plot between the two observables for the transformed and trained data.

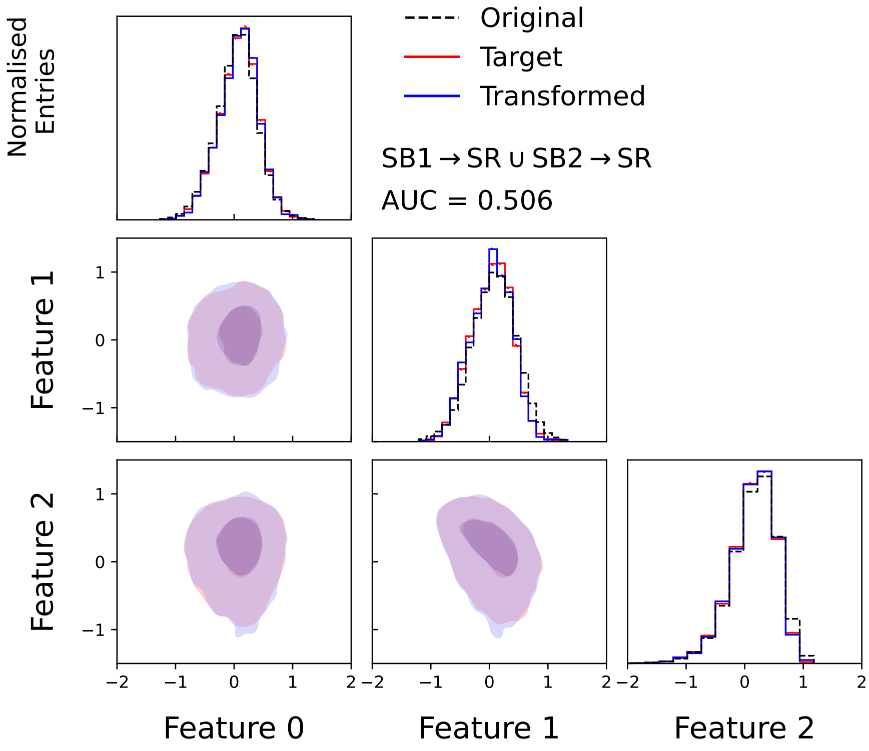

Figure S4. Input, target and transformed data distributions for a toy example with enhanced correlation between the input variables and the invariant mass. Features 0 and 1 are linearly correlated to m_{JJ} whilst and Feature 3 is proportional to the inverse cube of m_{JJ}. In comparison to the architecture optimised for the nominal features, the size of each coupling layer has been increased to four residual blocks, each with two hidden layers of 64 nodes. Distributions are shown for transforming data from SB1 and SB2 to the signal region to create the background template, with the model trained on SB1 (3200 $\leq m_{JJ} <$ 3400 GeV) and SB2 (3600 $\leq m_{JJ} <$ 3800 GeV). The data from SB1 (SB2) is transformed with a forward (inverse) pass of the CURTAINs model into the target region. The diagonal elements show the individual features with the off diagonal elements showing a contour plot between the two observables for the transformed and trained data.

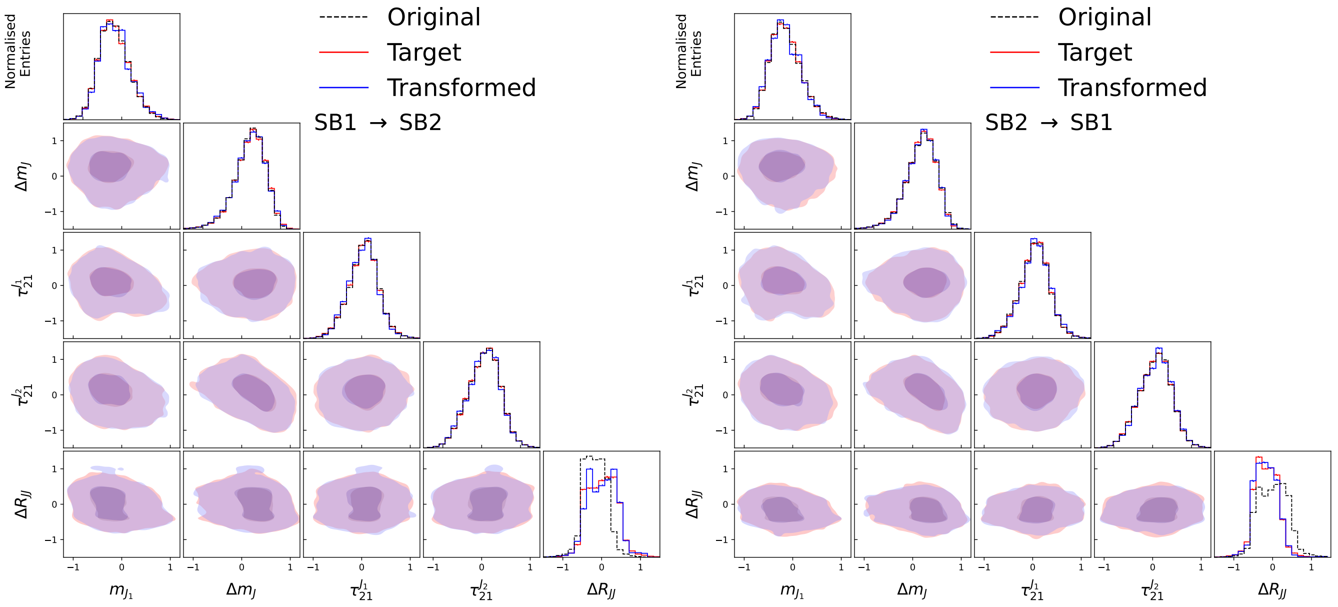

Figure S5. Input, target and transformed data distributions for the base variable set with the addition of ΔR_{JJ}, for transforming data from SB1 to SB2 (left) and SB2 to SB1 (right), with the model trained on SB1 ($3200 \leq m_{JJ} < 3400$ GeV) and SB2 ($3600 \leq m_{JJ} < 3800$ GeV). The data from SB1 (SB2) is transformed with a forward (inverse) pass of the CURTAINs model into the target region. The diagonal elements show the individual features with the off diagonal elements showing a contour plot between the two observables for the transformed and trained data. Only 25% of the available data has been used to train the CURTAINs model. The model architecture optimised for the full available training dataset has been used without any optimisation.

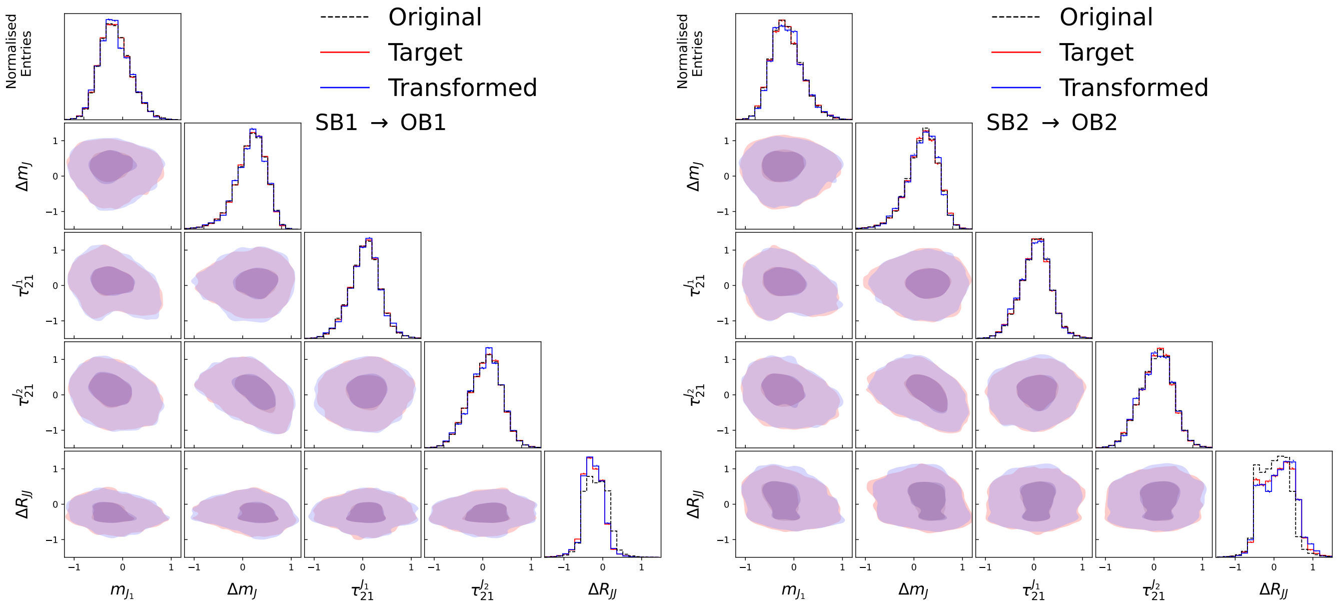

Figure S6. Input, target and transformed data distributions for the base variable set with the addition of ΔR_{JJ}, for transforming data from SB1 to OB1 (left) and SB2 to OB2 (right), with the model trained on SB1 ($3200 \leq m_{JJ} < 3400$ GeV) and SB2 ($3600 \leq m_{JJ} < 3800$ GeV), with OB1 and OB2 defined as 200 GeV wide windows directly next to SB1 and SB2 away from the signal region. The data from SB1 (SB2) is transformed with an inverse (forward) pass of the CURTAINs model into the target region. The diagonal elements show the individual features with the off diagonal elements showing a contour plot between the two observables for the transformed and trained data. Only 25% of the available data has been used to train the CURTAINs model. The model architecture optimised for the full available training dataset has been used without any optimisation.

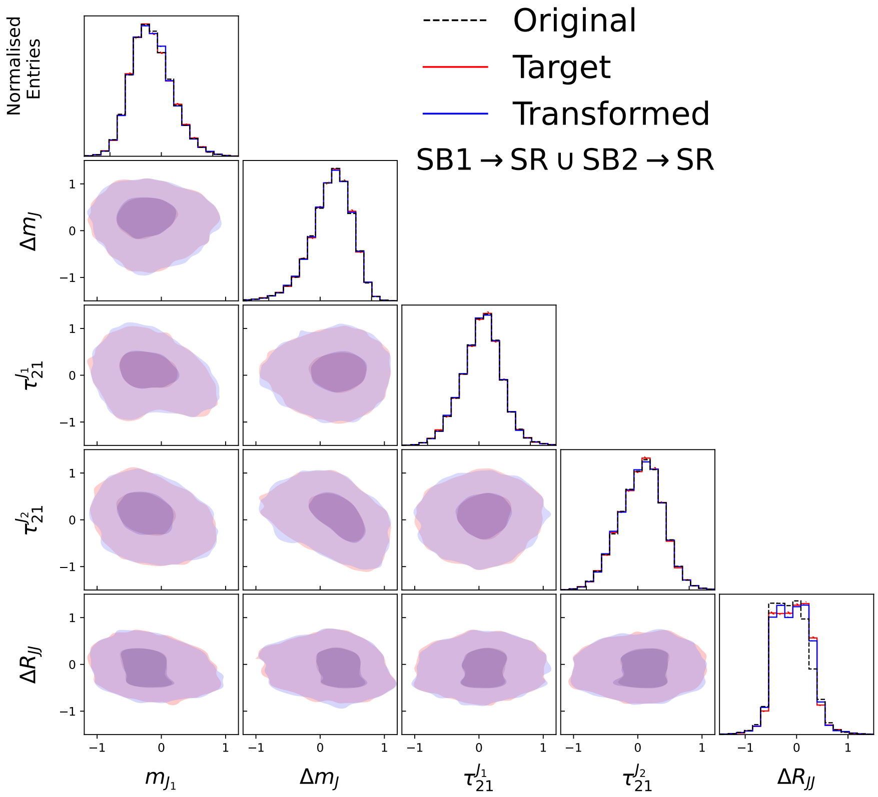

Figure S7. Input, target and transformed data distributions for the base variable set with the addition of ΔR_{JJ}, for transforming data from SB1 and SB2 to the signal region to create the background template, with the model trained on SB1 ($3200 \leq m_{JJ} < 3400$ GeV) and SB2 ($3600 \leq m_{JJ} < 3800$ GeV). The data from SB1 (SB2) is transformed with a forward (inverse) pass of the CURTAINs model into the target region. The diagonal elements show the individual features with the off diagonal elements showing a contour plot between the two observables for the transformed and trained data. Only 25% of the available data has been used to train the CURTAINs model. The model architecture optimised for the full available training dataset has been used without any optimisation.

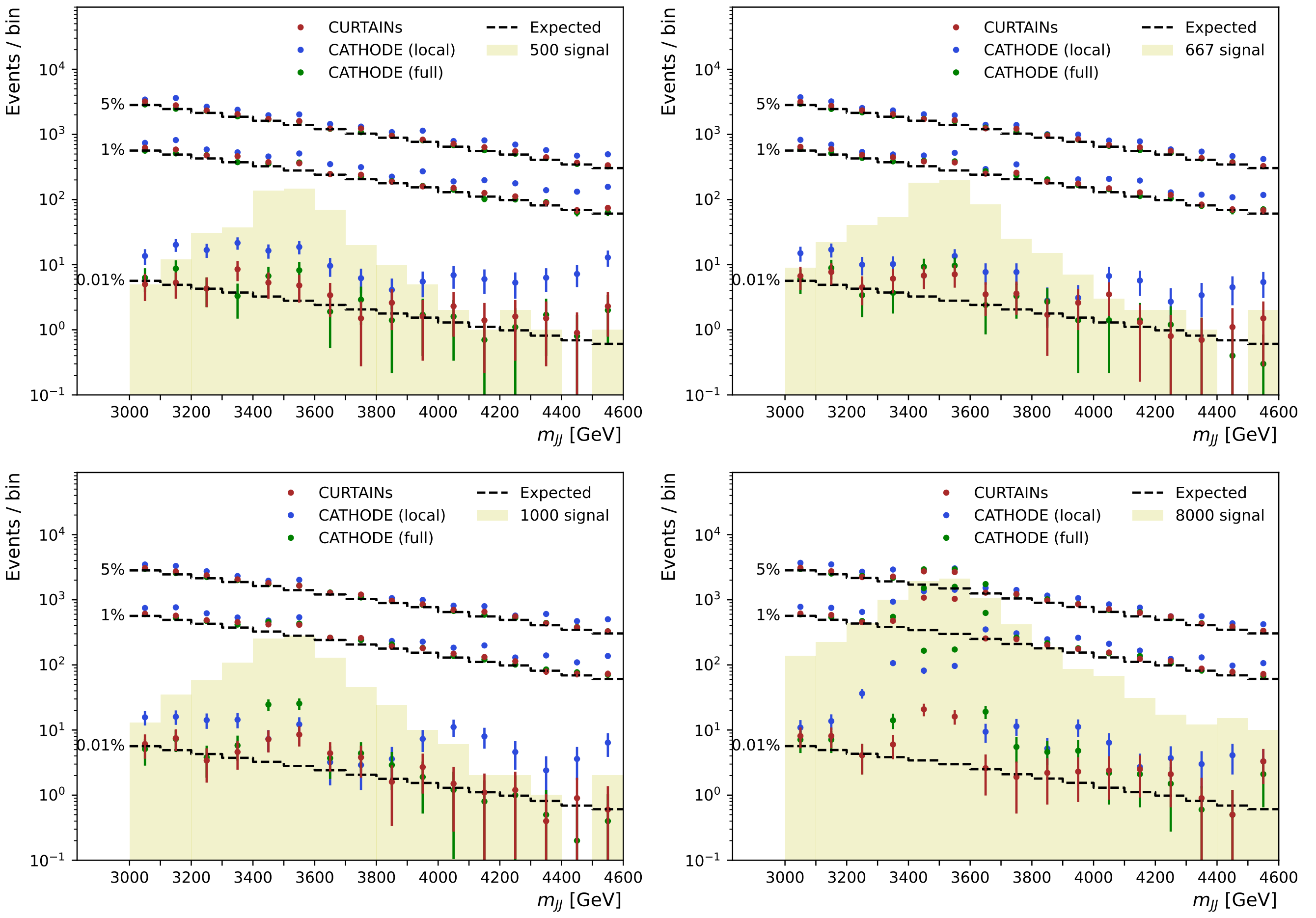

Figure S8. The dijet invariant mass for the range of signal regions probed in the sliding window, from 3300 GeV to 4600 GeV, for the case of samples doped with 500 (top left), 667 (top right), 1000 (bottom left) and 8000 (bottom right) signal events. Each signal region is 200 GeV wide and split into two 100 GeV wide bins . The dashed line shows the expected background after applying a cut on classifier trained using the background predictions from the CURTAINs (red), CATHODE (local) (blue) and CATHODE (full) (green) methods at specific background rejections. Three different cut levels are applied retaining 5%, 1% and 0.01% of background events respectively. The cut values are calculated per signal region using the background template.
